# Pathophysiology of Cisplatin-Induced Acute Kidney Injury

**DOI:** 10.1155/2014/967826

**Published:** 2014-08-06

**Authors:** Abdullah Ozkok, Charles L. Edelstein

**Affiliations:** Division of Renal Diseases and Hypertension, University of Colorado at Denver, P.O. Box C281, 12700 East 19th Avenue, Aurora, CO 80262, USA

## Abstract

Cisplatin and other platinum derivatives are the most widely used chemotherapeutic agents to treat solid tumors including ovarian, head and neck, and testicular germ cell tumors. A known complication of cisplatin administration is acute kidney injury (AKI). The nephrotoxic effect of cisplatin is cumulative and dose-dependent and often necessitates dose reduction or withdrawal. Recurrent episodes of AKI may result in chronic kidney disease. The pathophysiology of cisplatin-induced AKI involves proximal tubular injury, oxidative stress, inflammation, and vascular injury in the kidney. There is predominantly acute tubular necrosis and also apoptosis in the proximal tubules. There is activation of multiple proinflammatory cytokines and infiltration of inflammatory cells in the kidney. Inhibition of the proinflammatory cytokines TNF-*α* or IL-33 or depletion of CD4+ T cells or mast cells protects against cisplatin-induced AKI. Cisplatin also causes endothelial cell injury. An understanding of the pathogenesis of cisplatin-induced AKI is important for the development of adjunctive therapies to prevent AKI, to lessen the need for dose decrease or drug withdrawal, and to lessen patient morbidity and mortality.

## 1. Introduction

Acute kidney injury (AKI) is defined as a clinical syndrome characterized by a rapid decrease in renal function together with the accumulation of waste products such as urea [1]. The incidence of non-dialysis-requiring AKI is about 5000 cases per million people per year and incidence of dialysis requiring AKI is 295 cases per million people per year [2]. AKI complicates 1–7% of all hospital admissions and 1–25% of intensive care unit admissions [3, 4]. Furthermore, AKI is known as an independent risk factor for mortality. AKI increases the risk of death by 10- to 15-fold and results in a mortality rate of 50% [5, 6].

The kidneys are the major targets for the toxic effects of various chemical agents and thus drug-induced AKI is a frequent entity in clinical medicine. The incidence of nephrotoxic AKI is difficult to estimate due to variabilities of patient populations and criteria of AKI. However, nephrotoxicity has been reported to contribute to about 8–60% of hospital-acquired AKI cases [7]. In a recent large multicenter epidemiological survey performed on critically ill patients, drug nephrotoxicity was found to be responsible for 19% of AKI cases [8].

Cisplatin (dichlorodiamino platinum) is an inorganic platinum-based chemotherapeutic agent that is widely used in the treatment of a variety of solid malignant tumors such as head and neck, lung, testis, ovarian, and bladder cancers [9]. The use of cisplatin is frequently limited by various significant side effects such as bone marrow suppression, peripheral neuropathy, ototoxicity, anaphylaxis, and nephrotoxicity. After a single dose of cisplatin (50–100 mg/m^2^), approximately one-third of the patients develop nephrotoxicity [10, 11].

An improved knowledge of the pathogenesis of cisplatin-induced AKI is crucial to prevent the AKI and improve survival in cancer patients receiving cisplatin-based treatments. Furthermore, increased renal vascular resistance and decreases in renal plasma flow and glomerular filtration rate (GFR) make the cisplatin nephrotoxicity an ideal model to study the early pathophysiological features of all types of AKI [12]. In this paper, we aim to review the pathophysiological mechanisms of cisplatin-induced AKI and discuss the most recent experimental strategies and molecules to prevent cisplatin-induced AKI.

## 2. Cellular Uptake of Cisplatin

Uptake of the cisplatin by the renal cells is energy dependent and can be inhibited by probenecid [13]. Deletion of the Ctr1, a high-affinity copper transporter, results in reduced uptake of cisplatin and toxicity in yeast [14]. Ctr1 knockdown significantly reduced cisplatin-induced apoptosis in renal proximal tubular cells (RPTC) [15]. Organic cation transporters (OCTs) also play a role in cellular uptake of cisplatin [16]. Cimetidine, an OCT2 inhibitor, decreases cisplatin uptake in cultured renal tubular cells [17]. Cisplatin uptake and toxicity were shown to be increased in OCT2 overexpressing human proximal tubular cells [17]. However, OCT1/OCT2-deficient mice were protected from cisplatin-induced renal tubular damage [18, 19]. Cisplatin becomes highly reactive within the cell. Cisplatin conjugates with molecules such as glutathione, proteins, RNA, and DNA. Intra- and interstrand cross-linking of DNA by cisplatin block DNA replication and gene transcription. Thus, DNA damage is a critical component of cisplatin toxicity [20, 21].

Due to its low molecular weight and uncharged character, unbound cisplatin in the plasma is freely filtered by the glomerulus. Most of the cisplatin is trapped within the renal cortex [13, 22]. The concentration of cisplatin in the proximal tubular cells is 5 times higher than the serum concentration and thus such an accumulation of cisplatin in kidney contributes to its nephrotoxicity [9, 23].

## 3. General Pathophysiology

The pathophysiology of cisplatin-induced AKI involves 4 major mechanisms: (1) proximal tubular injury, (2) oxidative stress, (3) inflammation, and (4) vascular injury in the kidney. Proximal tubular injury involves several different mechanisms including apoptosis [24], autophagy [25], dysregulation of cell-cycle proteins [26], activation of the mitogen-activated protein kinase (MAPK) signaling pathways [27], direct toxicity to renal tubular epithelial cells [17], DNA damage [28], and mitochondrial dysfunction [29].

## 4. Proximal Tubular Injury

### 4.1. Apoptosis

The dosage of cisplatin determines whether the cells die by necrosis or apoptosis [30]. In cell culture studies, high concentrations of cisplatin cause necrotic cell death but lower concentrations lead to apoptosis. However,* in vivo*, cisplatin induces both necrosis and apoptosis [31]. Several pathways are defined for apoptosis such as the extrinsic pathway induced by death receptors, the intrinsic pathway depending mostly on mitochondria, and the endoplasmic reticulum (ER) stress pathway. In the extrinsic pathway, activation of death receptors such as Fas and tumor necrosis factor-alpha (TNFR) leads to activation of downstream caspases to induce apoptosis [32]. In the intrinsic pathway, cellular injury leads to the activation of the proapoptotic Bax and Bak proteins, which in turn results in the release of apoptogenic factors including cytochrome c, apoptosis-inducing factor (AIF), Smac/DIABLO, and endonuclease G from the mitochondria. Cisplatin induces Bax activation [33] and inhibition of Bax by Bcl-2 diminishes mitochondrial injury and cisplatin-induced apoptosis [34]. Importantly, when the Bax gene was deleted, the animals became resistant to cisplatin [24]. In the ER stress pathway of apoptosis, the initiator caspase is caspase-12 which was shown to be activated by cisplatin. In a cell culture study, apoptosis was found to be attenuated with transfection of an anti-caspase-12 antibody [35]. Another ER-associated protein during cisplatin injury is a calcium-independent phospholipase A2 (ER-iPLA2). Inhibition of ER-iPLA2 leads to amelioration of cisplatin-induced apoptosis in proximal tubular cultures [36]. In addition to caspases and proapoptotic pathways, there are other pathways like p53 that are activated in cisplatin-induced AKI.

#### 4.1.1. p53 and Apoptosis

In cultured renal tubular cells, p53 was shown to be activated by cisplatin [37]. Cisplatin induces DNA damage and subsequently DNA damage response proteins such as ataxia telangiectasia-mutated (ATM) proteins are activated leading to p53 phosphorylation. It was shown that both genetic and pharmacological inhibition (pifithrin) of p53 decreased the tubular cell apoptosis, renal tissue damage, and cisplatin-induced AKI [38].

Several p53-associated pathways and molecules have been defined such as p53 upregulated modulator of apoptosis (PUMA), histone deacetylase (HDAC) inhibitors, taurine transporter gene (TauT), and SIRT1 which are all implicated in the pathophysiology of cisplatin-induced AKI. PUMA, a proapoptotic protein, was identified as a major downstream mediator of the apoptotic actions of p53. PUMA is also induced by cisplatin [39]. This induction was shown to be dependent on p53 because PUMA was also inhibited by both pharmacological (pifithrin) and genetic ablation of p53. Furthermore, cisplatin-induced apoptosis was ameliorated in PUMA-knockout cells [39].

HDAC inhibitors such as suberoylanilide hydroxamic acid (SAHA) and trichostatin A (TSA) inhibit p53 phosphorylation, acetylation, and activation during cisplatin treatment. TSA and SAHA ameliorated the cisplatin-induced apoptosis in proximal tubular cells. HDAC inhibitors are also effective chemotherapeutic agents. The use of cisplatin and TSA in combined chemotherapy protocols may be an effective strategy to enhance the antitumor efficacy and to prevent cisplatin-induced nephrotoxicity [40].

Cisplatin was found to decrease the expression of the TauT through the activation of p53 in proximal tubular renal cells [41]. It was also shown that overexpression of TauT prevented the cisplatin-induced apoptosis and renal dysfunction in TauT transgenic mice. Functional TauT may prevent cisplatin-induced nephrotoxicity by attenuating p53-dependent pathway [41].

SIRT1 deacetylates p53 and in turn decreases apoptosis through deacetylation of p53 [42]. It has also been shown that kidney specific overexpression of SIRT1 was protective against cisplatin-induced AKI [43]. Resveratrol, a SIRT1 activator, reduced apoptosis and cytotoxicity in proximal tubular cells and also improved renal function* in vivo* [44].

In summary, there is activation of p53 in cisplatin-induced AKI and inhibition of p53 may decrease cisplatin-induced AKI. However, it should be noted that inhibition of p53 may also increase the survival of the cancer cells and thus reduce the therapeutic efficiency of cisplatin.

#### 4.1.2. p53-Independent Mechanisms and Antiapoptotic Proteins

p53-independent mechanisms of apoptosis have also been reported. In a study by Jiang et al. [45], cisplatin was capable of inducing Bax activation, cytochrome c release, and apoptosis even in primary cultures of p53-deficient renal tubular cells. Induced apoptosis was caspase dependent in this model and could be completely inhibited by general caspase inhibitors [45].

#### 4.1.3. Caspase-1 and Apoptosis

In the study by Faubel et al. [46], apoptosis, necrosis, inflammation, and renal dysfunction were all reduced in cisplatin-treated caspase-1−/− mice compared to wild-type mice. Specifically, caspase-3 activation preceded cisplatin-induced cell death and caspase-3 activity was significantly reduced in the cisplatin-treated caspase-1−/− mice. Thus caspase-1−/− mice are protected against cisplatin-induced AKI by a reduction in caspase-3 dependent apoptosis [46].

In summary, caspase inhibition protects cultured cells from cisplatin-induced apoptosis. The role of caspase-3 and apoptosis in cisplatin-induced AKI* in vivo* is more complex.* In vivo*, cisplatin causes extensive acute tubular necrosis (ATN) in addition to tubular apoptosis. Direct caspase-3 or apoptosis inhibition has not been reported to protect against cisplatin-induced AKI supporting the lack of a direct role of caspase-3-mediated apoptosis in causing the functional derangements in AKI. In fact, injection of a caspase inhibitor in mice worsened cisplatin-induced AKI [47]. In this study, caspase-3 inhibition in AKI was associated with less autophagy and worse AKI. In another study, decreases in renal apoptosis alone were not associated with functional protection against cisplatin-induced AKI as measured by BUN [48]. The role of autophagy in cisplatin-induced AKI is discussed in the next section.

### 4.2. Autophagy

Autophagy is a cellular process of “self-eating” in which cytoplasmic components are sequestered into autophagic vesicles and then delivered to lysosomes for degradation [49]. Autophagy has been demonstrated to be immediately induced in cisplatin-induced AKI [50]. The expression levels of markers of autophagy such as Beclin 1, LC3, and Atg5 were found to be significantly increased on exposure of RTEC to cisplatin [50]. Although some controversy exists about the role of autophagy in terms of cell survival [51], autophagy is considered as a renoprotective mechanism in cisplatin-induced AKI [50]. Autophagy was found to promote cellular survival and delay the onset of apoptosis in RTECs exposed to cisplatin [52]. In a recent study by Jiang et al. [53], chloroquine, a pharmacological inhibitor of autophagy, blocked autophagic flux and enhanced cisplatin-induced AKI. In contrast, rapamycin activated autophagy and protected against AKI. In addition, a renal proximal tubule-specific autophagy-related gene 7-knockout mouse model was found to be more sensitive to cisplatin-induced AKI compared to wild-type mice. These knockout mice had increased activation of p53 and c-Jun N terminal kinase [53]. In another study, use of pharmacological inhibitors of autophagy (3-methyladenine or bafilomycin) and shRNA knockdown of Beclin (an autophagic gene) was shown to increase cisplatin-induced tubular cell apoptosis [50].

There are other studies demonstrating that excessive or impaired autophagy may also lead to cell death [54]. In the study by Herzog et al. [47], zVAD-fmk, a pan-caspase inhibitor, blocked the clearance of the autophagosomal cargo leading to lysosomal dysfunction. Furthermore, zVAD-fmk worsened the cisplatin-induced AKI. Chloroquine, a lysomotropic agent which is known to impair autophagic flux, also exacerbated cisplatin-induced AKI [47].

Inhibition of other autophagy proteins like Beclin 1 may worsen cisplatin-induced RTEC injury. Beclin 1 is a unique autophagic gene involved in tumor suppression [55]. Beclin 1-mediated autophagy has a tumoricidal effect in contrast to its protective effect in cultured RTEC. Thus, Beclin 1 may not only prevent the cisplatin-induced nephrotoxicity; it may also enhance the antitumor activity of cisplatin [55].

In summary, autophagy may have a protective role in cisplatin-induced AKI and inhibition of autophagy may worsen AKI.

### 4.3. Cell Cycle, MAPK, and Other Pathways

#### 4.3.1. Cdk2-p21 Pathway

Cyclin-dependent kinase 2 (Cdk2) is a serine/threonine protein kinase that phosphorylates substrates for cell cycle progression [56]. Cisplatin cytotoxicity is dependent on Cdk2 activity [57]. Cdk2 knockout cells were resistant to cisplatin and these cells regained cisplatin sensitivity after transduction with wild-type Cdk2 [57]. Furthermore, purvalanol, a Cdk2 inhibitor, was found to have significant protective effects against cisplatin-induced AKI [57]. Cisplatin also induces upregulation of p21, a Cdk2 inhibitor [58]. p21 knockout mice were shown to have increased kidney cell cycle activity and increased cisplatin nephrotoxicity [26]. On the other hand, p21 overexpression and cell cycle inhibitor drugs such as roscovitine were shown to completely protect proximal tubular cells from cisplatin-induced apoptosis [59]. In addition to p21, p27 is another cyclin-dependent kinase inhibitor that induces cell cycle arrest. Sodium arsenite, by inducing p27, attenuated cisplatin-induced AKI in rats [60, 61]. In summary, Cdk2 is a mediator of cisplatin-induced tubular injury. Cell cycle inhibitors and the cyclin-dependent kinase inhibitors p21 and p27 protect against cisplatin-induced tubular injury.

#### 4.3.2. Mitogen-Activated Protein Kinase (MAPK) Pathways

MAPK pathways are cascades of serine/threonine kinases that regulate cell proliferation, differentiation, and survival [62]. Cisplatin is known to activate p38, ERK, and JNK/SAPK in renal epithelial cells [63]. In the study by Nowak et al. [64], ERK1/2 pathway was found to be activated by cisplatin in renal tubular cells. Inhibition of ERK1/2 with pharmacological MEK inhibitors such as PD98059 and U0126 improved cisplatin-induced mitochondrial dysfunction and apoptosis [64]. In the study by Jo et al. [27], inhibition of ERK1/2 pathway reduced TNF-alpha expression and caspase activation in kidney tissue. Furthermore,* in vivo*, ERK inhibition provided functional and histological protection in cisplatin-induced AKI. It may be concluded that ERK1/2 pathway is an upstream signal for TNF-alpha production and caspase activation in cisplatin-induced AKI [27]. The ERK 1/2 pathway has also been found to be related to tubular ciliary functions. Several recent studies have suggested a relationship between ciliary dysfunction and AKI [65]. In the study by Wang et al. [66], cilia became shorter during cisplatin treatment. These cilia-suppressed cells showed hyperactivation of ERK. Importantly, inhibition of ERK by U0126 preserved cilia during cisplatin treatment and protected against apoptosis.* In vivo*, U0126 prevented the loss of cilia from proximal tubules during cisplatin treatment and protected against AKI. It may be suggested that role of ERK in ciliary regulation is important in cisplatin-induced AKI [66]. There are other MAPK involved in the pathogenesis of cisplatin-induced AKI.

p38 is another important MAPK in the pathogenesis of cisplatin-induced AKI. Both* in vitro* and* in vivo*, cisplatin was shown to activate p38-MAPK [67].* In vivo*, SKF-86002, a p38-MAPK inhibitor, significantly decreased TNF-alpha levels and protected against cisplatin-induced AKI. Oxidative stress is known to activate p38-MAPK in kidney [68]. Also dimethylthiourea (DMTU), a hydroxyl radical scavenger, completely prevented cisplatin-induced AKI by the way of prevention of the activation of p38-MAPK [67].

In summary, cisplatin is known to activate p38, ERK, and JNK/SAPK in renal epithelial cells and inhibition of ERK1/2 improves cisplatin-induced mitochondrial dysfunction and apoptosis.

#### 4.3.3. Mammalian Target of Rapamycin (mTOR) Pathway

mTOR is a critical pathway for cellular survival, proliferation, protein synthesis, and autophagy [69]. Rapamycin, an mTOR inhibitor, has already been shown to protect against cisplatin-induced AKI by the way of activation of autophagy [53]. However mTOR kinase has two distinct protein complexes named mTOR complex 1 (mTORC1) and mTOR complex 2 (mTORC2). Rictor provides the assembly of mTORC2 and the interaction of mTORC2 with its substrates and regulators [70]. Tubule-specific deletion of Rictor (Tubule-Rictor−/−) in mice exacerbated cisplatin-induced AKI compared to that in the control littermates. In kidneys of the knockout mice, significantly less autophagy, less Akt phosphorylation, and more apoptosis were observed. Confirming this finding, also* in vitro*, Rictor siRNA transfection caused more cisplatin-induced apoptosis but less cisplatin-induced autophagy. Metformin, an inducer of autophagy, blunted increased apoptosis induced by Rictor siRNA. All these findings suggest that endogenous Rictor/mTORC2 protects against cisplatin-induced AKI, probably through Akt signaling and induction of autophagy [71].

#### 4.3.4. Peroxisome Proliferator-Activated Receptors (PPARs) Pathway

PPARs have critical roles in regulating lipid and glucose metabolism, cell growth, and differentiation [72]. PPAR-alpha has also been shown to have a significant anti-inflammatory and antiapoptotic activity [73]. Cisplatin reduces the transcriptional activity of PPAR-alpha and in turn fatty acid oxidation (FAO) enzymes are inhibited in kidney tissue [74]. Subsequent failure of oxidation of long-chain fatty acids and long-chain acyl-carnitines during cisplatin-induced AKI results in their accumulation and cellular toxicity [74]. Administration of bezafibrate, a known PPAR-alpha ligand, prevented the inhibition of FAO and the accumulation of toxic fatty acids in kidneys. Also fibrates ameliorated apoptotic and necrotic proximal tubular cell death, resulting in significant protection of renal function. However fibrate-induced protection against AKI was ameliorated in PPAR-alpha knockout mice [75]. Importantly, transgenic mice overexpressing PPAR-alpha in the proximal tubule were protected from cisplatin-induced AKI [76]. Similarly, WY-14643, a fibrate class of PPAR ligand, significantly suppressed cisplatin-induced cytokine/chemokine expression and thus prevented neutrophil accumulation and this drug also ameliorated cisplatin-induced AKI. In contrast, WY-14643 could not prevent inflammation and cisplatin-induced AKI in PPAR null mice [77]. In summary, induction of PPARs is protective against cisplatin-induced tubular injury.

## 5. Inflammation

### 5.1. Cytokines

The proinflammatory nature of cisplatin-induced AKI has been well documented [78]. Secretion of various cytokines such as IL-1-beta, IL-6, IL-18, monocyte chemotactic protein-1 (MCP-1), regulated upon activation normal T cell expressed and secreted (RANTES), macrophage inflammatory protein-2 (MIP-2), intercellular cell adhesion molecule-1 (ICAM-1), and transforming growth factor beta (TGF-beta) has been shown to be increased in cisplatin-induced AKI [79]. In the study by Faubel et al., cisplatin-induced AKI was associated with increases in the cytokines IL-1, IL-18, and IL-6; however, inhibition of IL-1, IL-18, and IL-6 could not prevent cisplatin-induced AKI [79].

Caspase-1 is a proinflammatory cytokine. Caspase-1 converts IL-1-beta and IL-18 to their active forms. Caspase-1−/− mice have a reduction in these proinflammatory mediators because of the lack of the mature IL-1b and IL-18 in response to cisplatin [46]. However, protection against cisplatin-induced AKI in caspase-1−/− mice is independent of IL-1b and IL-18 because inhibition of IL-1b or IL-18 does not protect against cisplatin-induced AKI [79]. Another proinflammatory cytokine, TNF-alpha, plays a central role in the pathogenesis of cisplatin-induced AKI [80].

### 5.2. TNF-Alpha

Cisplatin increases both serum and urine concentrations of TNF-alpha [80, 81]. Genetic or pharmacological inhibition of TNF-alpha reduces the expression of other inflammatory cytokines and chemokines such as IL-1-beta, MCP-1, and RANTES in cisplatin models [80]. Importantly, pharmacological inhibitors of TNF-alpha (GM6001 and TNF-neutralizing antibody) protected against cisplatin-induced AKI. Also TNF-alpha knockout mice were resistant to cisplatin nephrotoxicity [80]. Salicylates, by the way of TNF-alpha inhibition, have also been reported to be protective against cisplatin-induced AKI [82]. However salicylates had no beneficial effect in TNF-alpha knockout mice [82]. In another study by Kim et al. [83], pentoxifylline, a TNF-alpha inhibitor, protected against cisplatin nephrotoxicity* in vivo* [83].

In a study by Zhang et al. [84], to determine the contributions of renal parenchymal cells and bone marrow-derived cells to the pathogenesis of cisplatin-induced AKI, chimeric mice in which the bone marrow was ablated and replaced with donor bone marrow cells from wild-type or from TNF-alpha knockout mice were used. Chimeras with TNF-alpha knockout kidneys showed significantly less serum TNF-alpha levels and cisplatin-induced AKI regardless of the immune cell source. It may be concluded that local production of TNF-alpha by resident kidney cells rather than by bone marrow-derived infiltrating immune cells is crucial in cisplatin-induced AKI [84]. However there is controversy in this field. In another study by Liu et al. [81], deletion of T cells reduced TNF-alpha in kidneys and also protected against cisplatin-induced AKI suggesting an important role of bone marrow-derived immune cells in TNF-alpha production.

Two different receptors, TNFR1 and TNFR2, mediate the biological activities of TNF-alpha. TNFR1 mediates many cytotoxic and proinflammatory actions of TNF-alpha [85]. However TNFR2-deficient mice developed significantly less severe renal dysfunction compared to TNFR1-deficient mice. Furthermore, renal and serum TNF-alpha levels were lower in TNFR2-deficient mice. These results suggest that TNFR2 is more important in cisplatin-induced TNF-alpha production and AKI compared to TNFR1 [31].

### 5.3. Interleukin-33

IL-33 is a proinflammatory cytokine [86]. IL-33 is also a chemoattractant for CD4+ T cells via its receptor, ST2R [87]. When soluble ST2 (sST2) was injected as a decoy receptor to block IL-33 to cisplatin-treated mice, sST2-treated mice were found to have less CD4+ T cells infiltration and reduced acute tubular necrosis (ATN) and apoptosis in the kidney. Furthermore, sST2 protected against cisplatin-induced AKI. It should be noted that administration of recombinant IL-33 (rIL-33) exacerbated cisplatin-induced AKI in wild-type mice, however not in CD4-deficient mice, suggesting that CD4+ T cells mediated the injurious effects of IL-33. Wild-type mice that received cisplatin and rIL-33 also had higher levels of the chemokine CXCL1. Mice deficient in the CXCL1 receptor also had lower serum creatinine, ATN, and apoptosis compared to wild-type mice following cisplatin administration. Taken together, it may be concluded that IL-33 promotes AKI through CD4+ T cell-mediated production of CXCL1 [88].

### 5.4. Interleukin-10

IL-10 is an anti-inflammatory cytokine produced mainly by Th2 cells, regulatory T cells (Tregs), dendritic cells, and macrophages [89]. IL-10 inhibits the production of proinflammatory cytokines and chemokines. In general, dendritic cells have anti-inflammatory features by various mechanisms including the production of IL-10 [90]. In the study by Tadagavadi and Reeves [91], after cisplatin administration, IL-10 knockout mice had higher BUN and creatinine levels compared to wild-type mice. Furthermore, mixed bone marrow chimeric mice lacking IL-10 in dendritic cells showed worse renal dysfunction compared to chimeric mice with IL-10 in dendritic cells. Considering these data, it may be suggested that endogenous IL-10 has protective effects against cisplatin nephrotoxicity. Moreover, IL-10 production by dendritic cells is an important component of protective effects of dendritic cells in cisplatin-induced AKI [91].

### 5.5. Signaling Pathways That Activate Proinflammatory Cytokines

Cisplatin induces the phosphorylation and subsequent translocation of nuclear transcription factor-kappa B (NF-*κ*B) to the nucleus, through the degradation of the inhibitory protein I*κ*B*α* [92]. Within the nucleus, activated NF-*κ*B leads to transcription of inflammatory mediators including TNF-alpha [93]. In turn, TNF-alpha induces the expression of other inflammatory cytokines and recruitment of inflammatory cells into the kidney tissue [19]. The role of NF-*κ*B in cisplatin-induced AKI remains to be studied.

Besides the NF*κ*B pathway, other inflammatory pathways have been defined in the pathophysiology of cisplatin-induced AKI such as poly(ADP-ribose) polymerase-1 (PARP-1) and toll-like receptors (TLRs) pathways. PARP-1 has regulatory effects on various inflammatory genes, including TNF-alpha, IL-1-beta, IL-6, ICAM-1, and TLR4 [94]. PARP-1 inhibition or gene deletion is known to have renoprotective effects against ischemia/reperfusion induced AKI [95]. Pharmacological inhibition of PARP-1 by the drug PJ-34 significantly decreased inflammation after cisplatin injection and protected against cisplatin-induced AKI [96].

The TLR pathway plays a role in cisplatin-induced RTEC injury. TLRs have various functions as an important component of innate immune system by recognizing pathogen-associated molecular patterns and also endogenous signals of tissue injury [97]. Renal toxicity of cisplatin has been demonstrated to be TLR4-dependent [93]. Mice with targeted deletion of TLR4 [Tlr4(−/−)] had significantly less leukocyte infiltration and cisplatin-induced AKI. Levels of cytokines in serum, kidney, and urine were also significantly reduced in Tlr4(−/−) mice compared to wild-type mice. Activation of JNK and p38 pathways was also blunted in Tlr4(−/−) mice. It was also demonstrated that renal parenchymal TLR4, rather than myeloid TLR4, mediated the cisplatin-induced nephrotoxicity. It may be concluded that, in cisplatin-induced AKI, activation of TLR4 on renal parenchymal cells may activate p38-MAPK pathways leading to nephrotoxicity [98].

In summary TNF-alpha and IL-33 are proinflammatory cytokines that mediate cisplatin-induced AKI while IL-10 is an anti-inflammatory cytokine that protects against cisplatin-induced AKI. The pathways that lead to cytokine production like NF*κ*B, PARP-1 remain to be studied in detail in cisplatin-induced AKI.

### 5.6. Inflammatory Cells of the Immune System

Inflammatory cells of the immune system such as T cells, macrophages, neutrophils, and mast cells are known to infiltrate the kidney tissue and play a role in the development of cisplatin-induced AKI. A pathophysiological role for T lymphocytes, especially CD4+ T cells, has been well established in cisplatin-induced AKI [81]. However, the role of the other inflammatory cells is less well defined. For example, in the study by Faubel et al. [79], by the use of neutrophil-depleting antibody, RB6-8C5, renal neutrophils were depleted in a cisplatin-induced AKI model but renal function or tubular necrosis was not improved [79].

Adhesion molecules such as ICAM (CD54) are important for leukocyte recruitment to the inflamed tissues. The importance of ICAM in the pathogenesis of the cisplatin-induced AKI was investigated in a study using an anti-CD54 antibody [99]. An improvement in renal function and mortality was observed in animals treated with anti-CD54 antibody in this cisplatin-induced AKI model [99].

#### 5.6.1. T Cells

Liu et al. [81] reported that CD4 T cell-deficient mice had significantly better renal function compared to wild-type controls in cisplatin-induced AKI. Adoptive transfer of CD4+ T cells into these T cell-deficient mice resulted in increased renal injury. CD4- and CD8-deficient mice were also protected from cisplatin-induced AKI compared with wild-type mice [81].

Activated T lymphocytes express Fas ligand (FasL) and T cell immunoglobulin mucin 1 (Tim-1) that may be important for T cell mediated cytotoxicity. Until recently, the FasL- associated apoptotic stimulus was thought to be mediated only through the T lymphocytes [81]. However, in the study by Linkermann et al. [100], for the first time, primary tubular cells were shown to mediate FasL-mediated cell death in the complete absence of immune cells. Treating severe combined immunodeficiency (SCID)/beige mice with MFL3, a FasL-blocking monoclonal antibody, completely restored survival after an otherwise lethal cisplatin dose, suggesting another source of FasL besides immune cells. Thus, cisplatin-induced AKI may be mediated through FasL expressed on tubular cells rather than inflammatory cells [100]. Besides activated T cells [101], Tim-1 is also expressed by tubular epithelial cells after ischemic or toxic injury and it is called kidney injury molecule-1 (Kim-1) [102]. In a cisplatin nephrotoxicity model, anti-Tim-1 antibodies were found to reduce renal NF-*κ*B activation, inflammation, and CD4 and CD8 T cell activation and apoptosis [103]. Furthermore, anti-Tim-1 antibodies significantly attenuated cisplatin-induced AKI. Renal Kim-1 expression was also reduced with the use of anti-Tim-1 antibodies [103].

#### 5.6.2. T-Regulatory Cells

Currently available evidence indicates that CD4+CD25+ Treg cells can suppress innate immunity and CD4+ T cell-mediated pathology in the kidneys [104]. Recent studies have also suggested that CD4+CD25+ Treg cells can inhibit activation of macrophages ameliorating the glomerular and interstitial injury in adriamycin nephropathy [105]. In a recent study by Lee et al. [106], CD4+CD25+ Treg cells were injected to the nu/nu mice that lack mature T cells and possible protective effects of the CD4+CD25+ Treg cells against cisplatin-induced AKI were investigated. In this model, CD4+CD25+ Treg cells were found to significantly improve the survival and renal functions in these mice. Furthermore, renal macrophage recruitment was also decreased in CD4+CD25+ Treg cell-adoptive transferred nu/nu mice compared with control mice. CD4+CD25+ Treg cells were also protective against cisplatin-induced AKI in wild-type Balb/c mice. Consistently, depletion of CD4+CD25+ Treg cells in wild-type mice exacerbated cisplatin-induced AKI [106]. Thus Treg cells play a protective role in cisplatin-induced AKI.

#### 5.6.3. Macrophages

Cisplatin-induced AKI is associated with increased renal myeloperoxidase, which is produced by both neutrophils and macrophages [46]. Renal infiltration of macrophages was shown to occur late in the course of cisplatin-induced AKI [107]. Macrophage depletion by liposome-encapsulated clodronate resulted in an effective decrease in renal CD11b-positive macrophages. However, macrophage depletion did not protect against cisplatin-induced AKI. Fractalkine (CX3CL1) is expressed by injured endothelium and functions as a potent chemoattractant for macrophages [108]. To study the role of CX3CR1, both anti-CX3CR1 antibody and CX3CR1−/− mice were used; however, these strategies were also not protective against cisplatin-induced AKI [109].

#### 5.6.4. Mast Cells

Mast cells are capable of secreting cytokines and chemokines essential for leukocyte recruitment and adhesion [110]. Mast cells are also unique because they have preformed TNF-alpha which can be released immediately after degranulation [111]. Summers et al. [112] used KitW-sh/W-sh mice to study mast cell deficiency in cisplatin-induced AKI. These mice were found to have decreased renal injury, serum TNF-alpha levels, and reduced leukocyte recruitment compared to wild-type mice. When mast cell-deficient mice were reconstituted with mast cells from wild-type mice, increased TNF-alpha levels and cisplatin-induced renal dysfunction were observed similar to wild-type mice. However, mast cell-deficient mice reconstituted with mast cells from TNF-alpha-deficient mice remained to be protected against cisplatin-induced AKI. Furthermore, mast cell stabilizer, sodium chromoglycate, also significantly ameliorated renal injury in cisplatin-induced AKI. In summary, these results suggest that mast cells mediate cisplatin-induced AKI through the production of TNF-alpha [112].

#### 5.6.5. Dendritic Cells

Dendritic cells (DC) have anti-inflammatory and immune-tolerance inducing features by the way of production of TGF-beta, IL-10, clonal deletion of autoreactive T cells, and induction of Treg cells [113–115]. DC-depleted mice had more severe renal dysfunction, tubular injury, neutrophil infiltration, and higher mortality compared to nondepleted mice [90]. IL-10 production by dendritic cells was found to be an important component of the protective effects of dendritic cells in cisplatin-induced AKI [108]. In another study, depletion of DCs in the same model did not result in worse AKI [116].

In summary, depletion of CD4+ T cells or mast cells, but not neutrophils and macrophages results in protection against cisplatin-induced AKI. Treg cells and DCs may play a protective role in cisplatin-induced AKI.

## 6. Oxidative Stress

Generation of reactive oxygen species (ROS), accumulation of lipid peroxidation products in kidneys, and suppressed antioxidant systems are thought to be major mechanisms of cisplatin-induced AKI [117]. Within the cell, cisplatin is converted into a highly reactive form rapidly reacting with thiol-containing antioxidant molecules such as glutathione [20]. Consequently, depletion of glutathione leads to increased oxidative stress within the cells. Cisplatin may also cause mitochondrial dysfunction and increased ROS production through an impaired respiratory chain [118]. Finally, cisplatin may induce ROS formation via the cytochrome P450 (CYP) system [119]. In CYP2E1-null mice, cisplatin-induced ROS accumulation was attenuated and CYP2E1-null mice were protected against cisplatin-induced AKI [120].

There are inhibitors of oxidant stress that protect against cisplatin-induced RTEC injury. Sodium thiosulfate [121] and metabolites of amifostine (WR-2721) have been known for a very long time to inactivate toxic platinum species and protect against cisplatin nephrotoxicity [122]. Amifostine decreases nephrotoxicity probably by donating antioxidant thiol groups [123, 124]. Hydroxyl radical scavengers such as dimethyl sulfoxide (DMSO), mannitol, and benzoic acid significantly reduced cisplatin-induced cytotoxicity. Furthermore, both DMSO and dimethylthiourea (DMTU) were significantly protective against cisplatin-induced AKI in* in vivo* models [125]. DMTU was also shown to inhibit the p38-MAPK pathway, Fas, FasL, and TNF-alpha [126]. DMTU and N-acetylcysteine (NAC) suppressed hydroxyl radical accumulation, p53 activation, and cisplatin nephrotoxicity both* in vitro* and* in vivo* [127]. NAC was also demonstrated to inhibit NF-*κ*B, TNF-alpha, and p38-MAPK-mediated apoptosis and renal dysfunction in cisplatin-treated rats [128]. Vitamin C and vitamin E were also found to be renoprotective in cisplatin-treated mice [129]. Edaravone, a free radical scavenger, has demonstrated to have cytoprotective properties in murine proximal tubular cells [130].

### 6.1. Iron Metabolism and Oxidative Stress

Iron plays an important role in oxidative stress mediated tissue injury in cisplatin-induced nephrotoxicity. To investigate the role of cytochrome P-450 as a source of catalytic iron in cisplatin-induced AKI, piperonyl butoxide, a cytochrome P-450 inhibitor, was administered in cisplatin nephrotoxicity model [119]. Cisplatin significantly decreased cytochrome P-450 content and increased bleomycin-detectable iron in the kidney. However, piperonyl butoxide prevented cisplatin-induced loss of cytochrome P-450 as well as iron accumulation in the kidney. Importantly, piperonyl butoxide also ameliorated cisplatin-induced AKI [119]. Iron chelators such as deferoxamine and 1,10-phenanthroline were found to significantly attenuate the cisplatin-induced cytotoxicity. Notably, deferoxamine was found to have protective effects against cisplatin-induced AKI in rats [125].

### 6.2. Heme Oxygenase-1 (HO-1) Pathway and Oxidative Stress

HO-1 is a redox-sensitive microsomal enzyme that catalyzes the degradation of heme into biliverdin, iron, and carbon monoxide [131]. HO-1 is known to be activated in the kidney by the cisplatin treatment [132]. HO-1 knockout mice were markedly more sensitive to cisplatin-induced AKI. Furthermore, overexpression of HO-1 significantly ameliorated cisplatin-induced apoptosis [11]. In proximal tubular cell cultures, HO-1 knockout cells had higher levels of basal autophagy, impaired progression of autophagy, and increased apoptosis with cisplatin administration. Restoring HO-1 expression in these cells reversed the autophagic response and decreased the cisplatin-induced apoptosis. In addition, cells with overexpression of HO-1 had significantly lower levels of ROS and these cells were also protected from cisplatin cytotoxicity [133].

### 6.3. NADH: Quinone Oxidoreductase-1 (NQO1) and Oxidative Stress

NADH: quinone oxidoreductase 1 (NQO1) is an antioxidant flavoprotein that increases intracellular NAD+ levels [155]. Moreover, NQO1 has various functions such as activation of anti-inflammatory processes, scavenging of superoxide anion radicals, and stabilization of p53 [156]. Beta-lapachone is identified as an activator of NQO1 [157]. Beta-lapachone increased the intracellular NAD+/NADH ratio in renal tissues treated with cisplatin. Also inflammatory cytokines and AKI were significantly ameliorated by beta-lapachone. Importantly, beta-lapachone had no protective effect in NQO1−/− mice treated with cisplatin. Furthermore, beta-lapachone potentiated the tumoricidal action of cisplatin in a xenograft tumor model [137].

In summary, generation of ROS plays a role in mediating cisplatin-induced AKI. Antioxidant molecules like HO-1 and NQO1 are protective against cisplatin-induced AKI.

## 7. Renal Hemodynamics and Vascular Injury

Renal vasoconstriction caused by endothelial dysfunction and impaired vascular autoregulation is an important component of the pathophysiology of cisplatin-induced AKI. Cisplatin has been shown to induce acute ischemic damage with a reduction in medullary blood flow resulting in tubular cell injury [158]. Instead of usual autoregulatory renal vasodilatation that occurs in ischemic kidney, a marked vasoconstriction develops in cisplatin-induced AKI causing further hypoxic injury [159]. Cisplatin decreases effective renal plasma flow before any alteration in the GFR in humans [160]. Similarly, in rats, renal blood flow has been demonstrated to be reduced 2-3 days after cisplatin administration [158]. These renal hemodynamic alterations may be associated with an increase in cytosolic calcium in the glomerular arterioles [161]. Consistently, calcium channel blockers were shown to reverse the renal vasoconstriction and attenuate cisplatin-induced renal dysfunction [162]. Another possible cause of renal vasoconstriction induced by cisplatin is the reduced COX-2 and vasodilatatory prostaglandins [163].

Cisplatin is directly toxic to endothelial cells. In a study by Dursun et al., cultured pancreatic microvascular endothelial (MS1) cells were exposed to low and high concentrations of cisplatin. Cells treated with low concentration of cisplatin had normal ATP levels, increased caspase-3 activity, and apoptosis. However, cells treated with higher concentration of cisplatin had severe ATP depletion, increased caspase-3 activity, and necrosis. Calpain activity significantly increased with higher concentrations of cisplatin. Both pan-caspase inhibitor and calpain inhibitor were able to reduce cisplatin-induced necrosis. It was demonstrated that, in cisplatin-treated endothelial cells, caspases can also cause necrosis. Furthermore, calpain inhibitors may protect the endothelial cells from necrosis independent of caspase-3 [164].

In clinical practice, various types of cisplatin-induced vascular toxicities can be caused by cisplatin such as thrombotic microangiopathy and myocardial infarction. The vascular endothelial injury is an important component of cisplatin-induced AKI [166]. von Willebrand factor (vWF) is synthesized by endothelial cells, and increased plasma vWF levels are associated with endothelial cell injury [167]. In the study by Lu et al. [109], circulating vWF levels were increased after cisplatin administration with the peak vWF level preceding the renal dysfunction.

## 8. Treatment of Cisplatin-Induced AKI

Although many experimental therapies have been developed for prevention and treatment of cisplatin-induced AKI, current clinical practice only includes supportive measures while waiting for renal function to recover (Table 1). In this review, many of the experimental therapies and their corresponding pathophysiological pathways have been discussed. Pathophysiological pathways implicated in cisplatin-induced AKI and their* in vivo* therapeutic models and molecules are presented in Table 2. Herein we will discuss the general measures to prevent cisplatin-induced AKI and regenerative treatment strategies.

## 9. General Measures to Prevent Cisplatin-Induced AKI

The most important supportive measures are hydration, replacement of electrolyte losses, and avoidance of other potentially nephrotoxic drugs. Renal function (GFR) should be routinely assessed before each administration of cisplatin. Hydration should be started before the treatment and should be maintained for at least 3 days after the treatment. The adequacy of the hydration may be determined by the measurement of urine output which should be maintained at least at 3-4 L/day. Magnesium wasting is commonly seen in the course of cisplatin-induced AKI [168]; thus routine assessment of serum magnesium levels may be recommended in all patients receiving cisplatin treatment. Magnesium should be replaced adequately according to serum magnesium levels [22].

## 10. Regenerative Treatments

### 10.1. Erythropoietin

Erythropoietin (EPO) receptors have been demonstrated to be expressed on renal tubular cells [169]. For the first time in the literature, Vaziri et al. [145] reported that recombinant human EPO (rHuEpo) increased tubular regeneration and ameliorated cisplatin-induced AKI in rats. In another study, rHuEpo was shown to significantly reduce cisplatin-induced apoptosis in human renal proximal tubular epithelial (RPTE) cell culture [146]. Furthermore, tyrphostin AG-490, a JAK2 inhibitor, attenuated rHuEpo-induced protection against apoptosis, suggesting a role of the JAK-STAT pathway in rHuEpo-mediated antiapoptosis [146]. In a study by Bi et al. [147], EPO caused expansion and mobilization of mesenchymal stem cells (MSCs) from bone marrow to peripheral circulation in mice. Importantly, EPO ameliorated the cisplatin-induced AKI. Additionally, intraperitoneal injection of cultured EPO-mobilized cells resulted in improvement of cisplatin-induced AKI. It is possible that expansion and activation of MSCs may contribute to the renoprotective effects of EPO [147].

### 10.2. Mesenchymal Stem Cell (MSC) Transplantation

Bone marrow cells have been reported to differentiate into RTEC and contribute to regeneration of renal tubules after ischemic injury [170]. In the study by Iwasaki et al. [148], granulocyte-colony stimulating factor (G-CSF) was injected to BALB/c mice with cisplatin-induced AKI. G-CSF improved the survival of the mice and protected against AKI. BALB/c mice received bone marrow transplantation from enhanced green fluorescent protein (EGFP) transgenic mice. In the transplanted mice that were treated with G-CSF, the number of EGFP tubular epithelial cells was significantly higher in the kidney. It may be suggested that bone marrow cells mobilized by G-CSF may have protective effects against cisplatin-induced AKI [148].

MSCs are protective in mouse models of AKI [149]. MSCs, when injected intravenously, improved cisplatin-induced AKI and survival [150]. In a recent study, EPO-secreting MSCs (EPO gene-enhanced MSCs) were generated and then injected intraperitoneally to allogeneic mice with cisplatin-induced AKI. EPO-MSCs significantly improved survival and kidney function in this model. Furthermore, the presence of implanted cells in recipient kidneys was proved by PCR analysis. EPO-MSCs also significantly increased the expression of EPO and phosphorylated-Akt in these kidneys [151]. In the study by Yuan et al. [152], VEGF-modified human embryonic MSCs (VEGF-hMSCs) were cocultured with cisplatin-injured renal tubular epithelial cells. Improvement in the survival of tubular cells was observed via mitogenic and antiapoptotic actions. In addition, VEGF-hMSCs were also administered to a nude mouse model of cisplatin-induced AKI and these stem cells were shown to be protective against AKI [152].

The concept that the MSCs are able to transdifferentiate into tubular cells within the injured kidney and promote recovery is somewhat controversial. Actually, the homing of MSCs to sites of tissue injury and differentiation into tubular cells are very rare [171]. Beneficial effects of MSCs are mostly dependent on paracrine and endocrine actions such as immunomodulation and secretion of growth factors and cytokines [153, 171]. In the recent study by Kim et al. [154], adipose tissue derived MSCs (Ad-MSCs) ameliorated cisplatin-induced AKI. Activation of p53, JNK, and ERK pathways was hindered and apoptosis was significantly reduced by infusion of Ad-MSCs. However, very few Ad-MSCs could be detected within the kidney. Conditioned medium from cultured Ad-MSCs had also renal-protective functions both* in vivo* and* in vitro* [154]. In another study, hMSCs-conditioned media improved survival of human proximal tubular cells treated with cisplatin [172].

As mentioned previously, HO-1 is known to have important anti-inflammatory and antiapoptotic properties [173]. In the study by Zarjou et al. [165], MSCs were harvested from bone marrow of HO-1+/+ and HO-1−/− mice. Conditioned medium of HO-1+/+ MSCs had protective effects against cisplatin-induced AKI; however, the HO-1−/− conditioned medium was ineffective. HO-1 may be suggested to be important in MSCs-mediated protection of cisplatin-induced AKI [165].

## 11. Summary

Inflammation, proximal tubular, oxidative stress, and vascular injury are implicated in the pathogenesis of cisplatin-induced AKI. In the proximal tubules, there is predominantly not only acute tubular necrosis but also apoptosis. Inhibition of the proinflammatory cytokines TNF-*α* and IL-33 or depletion of CD4+ T cells or mast cells protects against cisplatin-induced AKI. Inhibitors of oxidative stress protect against cisplatin-induced AKI. Caspases and calpain play a role in cisplatin-induced injury to endothelial cells. Potential therapies for cisplatin-induced AKI based on pathophysiological mechanisms of injury include EPO (inhibits tubular apoptosis), MSCs, cytokine inhibitors (TNF-*α* or IL-33 inhibitors), inhibitors of the MAPK pathway, inhibitors of oxidant stress, and anti-inflammatory agents that can reduce CD4+ T cells.

## Figures and Tables

**Table 1:** General measures for prevention and treatment of cisplatin-induced AKI.

(1) Determine renal function (GFR) before each session of cisplatin treatment	
(2) Determine the risk of AKI (high risk; females, elderly patients, dehydration, patients with CKD and repeated doses of cisplatin)	
(3) Adjust cisplatin dose according to patient's renal function	
(4) Start hydration (with saline) before cisplatin and maintain for at least 3 days after treatment	
(5) Watch for electrolyte wasting (e.g., Mg), replace appropriately	
(6) Avoid concomitant nephrotoxic agents (NSAIDs, aminoglycosides, contrast agents, etc.)	
(7) Determine renal function within 1 week of treatment	
(8) Amifostine may be considered in patients with high risk of AKI	
(9) Consider newer, less nephrotoxic platinums such as carboplatin and oxaliplatin	

GFR: glomerular filtration rate, AKI: acute kidney injury, CKD: chronic kidney injury, Mg: magnesium, and NSAIDs: nonsteroid anti-inflammatory drugs.

**Table 2:** Pathophysiological pathways implicated in cisplatin-induced AKI and their corresponding *in vivo* protective models and molecules.

Pathophysiological pathway	Protective model/molecule	Effect	References
Apoptosis	Pifithrin	p53 inhibition—decreased apoptosis	[38]
	p53 knockout mice	p53 inhibition—decreased apoptosis	[38]
	Bax knockout mice	Bax inhibition—decreased apoptosis	[24]
	SIRT-1 overexpression	Deacetylation of p53—decreased apoptosis	[43]
	Resveratrol	SIRT-1 activator—decreased apoptosis	[44]
	Taurine transporter gene (TauT) transgenic mice	p53 inhibition—decreased apoptosis	[134]

Autophagy	Rapamycin	Induction of autophagy	[53]

ERK pathway	U0126	MEK-ERK inhibition	[27, 66]

p38 MAPK pathway	SKF-86002	p38 MAPK inhibition	[67]

Protein kinase C gamma pathway	PKC*δ* knockout model	Decreased apoptosis	[135]
	Rottlerin	PKC*δ* inhibition—decreased apoptosis	[135]

PPAR pathway	WY-14643 (PPAR ligand-fibrate)	PPAR activation	[136]
	PPAR transgenic mice	Increased PPAR activity	[76]

Oxidative stress	N-Acetylcysteine	Antioxidant	[128]
	Dimethylthiourea	Hydroxyl radical scavenging	[67, 68, 125, 127]
	Dimethyl sulfoxide	Hydroxyl radical scavenger	[125]
	b-Lapachone	NQO1 activator	[137]
	Amifostine	Antioxidant	[122, 123]
	Sodium thiosulfate	Antioxidant	[121]
	Vitamins C and E	Antioxidant	[129]

Cell cycle	E2F1 knockout mice	Decreased apoptosis	[138]
	Purvalanol	Cdk2 inhibitor—decreased apoptosis	[57]
	Sodium arsenite	p27 induction—decreased apoptosis	[60]

Mitochondrial metabolism	MDIVI-1	Dynamin-related protein-1 inhibition	[139]
	PKG-1 overexpressing transgenic mice	Improvement of mitochondrial functions—decreased apoptosis	[140]
	Sildenafil	PKG-1 activation	[140]

Iron metabolism	Cytochrome P450-2E1-null mice	Cytochrome P-450 inhibition	[120]
	Piperonyl butoxide	Cytochrome P-450 inhibition	[119]
	Deferoxamine	Iron chelation	[125]

Inflammation	GM6001	TNF-alpha inhibition	[80]
	TNF-alpha neutralizing antibody	TNF-alpha inhibition	[80]
	TNF-alpha knockout mice	TNF-alpha inhibition	[80]
	Salicylates	TNF-alpha inhibition	[82]
	Pentoxifylline	TNF-alpha inhibition	[83]
	Caspase-1 knockout	Decreased inflammation and apoptosis	[46]
	Anti-ICAM-1 (CD54)	Decreased neutrophil infiltration	[99]
	TLR4 knockout mice model	Decreased inflammation	[98]
	CXCR-2 knockout mice	Decreased inflammation	[88]
	Soluble ST2	Decreased inflammation, CD4+ T cell infiltration, and apoptosis	[88]

Prostaglandin metabolism	Microsomal prostaglandin E synthase-1 knockout mice	Decreased inflammation	[141]
	Celecoxib	COX-2 inhibition	[141]

T cells	T cell—deficient, CD4 and CD8 knockout models	Decreased inflammation	[81]
	Anti-Tim-1 antibodies	Decreased inflammation and apoptosis	[103]
	CD4+CD25+ Treg cells	Decreased inflammation and apoptosis	[106]

Mast cells	Mast cell-deficient mice model (KitW-sh/W-sh)	Decreased leukocyte infiltration and inflammation	[112]
	Sodium chromoglycate	Mast cell stabilization	[112]

Glucagon-like peptide-1 (GLP-1)	Exendin-4	GLP-1 receptor agonist—decreased apoptosis	[142]
	Alogliptin	Dipeptidyl peptidase-4 inhibition—decreased apoptosis	[142]

Klotho metabolism	Transgenic Klotho overexpressing (Tg-Kl) mice	Decreased apoptosis and decreased uptake of cisplatin	[143]

Metalloproteinase metabolism	Actinonin	Meprin A inhibitor	[144]

Poly-ADP-ribose polymerase-1 metabolism	PJ-34	PARP1 inhibition—decreased inflammation	[96]

Regeneration	Erythropoietin	Decreased apoptosis and increased mobilization of BM cells	[145–147]
	Granulocyte colony stimulating factor	Increased mobilization of BM cells	[148]
	Mesenchymal stem cells	Increased regeneration, decreased apoptosis	[149–154]

Anti-ICAM-1: anti-intercellular adhesion molecule-1, anti-TIM1: anti-T cell immunoglobulin mucin 1, BM: bone marrow, COX-2: cyclooxygenase-2, Cdk2: cyclin-dependent kinase-2, ERK: extracellular signal-regulated kinases, GLP-1: glucagon-like peptide-1, MAPK: mitogen-activated protein kinase, NQO1: NAD(P) H: quinone oxidoreductase 1, PARP-1: poly-ADP-ribose polymerase-1, PKC: protein kinase C, PKG-1: protein kinase G, PPAR: peroxisome proliferator-activated receptor, TLR-4: toll-like receptor 4, and TNF-alpha: tumor necrosis factor-alpha.
